# Ethanol Alters Alveolar Fluid Balance via Nadph Oxidase (NOX) Signaling to Epithelial Sodium Channels (ENaC) in the Lung

**DOI:** 10.1371/journal.pone.0054750

**Published:** 2013-01-29

**Authors:** Charles A. Downs, David Q. Trac, Lisa H. Kreiner, Amity F. Eaton, Nicholle M. Johnson, Lou Ann Brown, My N. Helms

**Affiliations:** 1 Nell Hodgson Woodruff School of Nursing, Emory University, Atlanta, Georgia, United States of America; 2 Department of Pediatrics, Emory-Children Center for Developmental Lung Biology, Emory University, Atlanta, Georgia, United States of America; 3 Emory Alcohol and Lung Biology Center, Emory University, Atlanta, Georgia, United States of America; 4 Department of Physiology, Emory University, Atlanta, Georgia, United States of America; University of Pittsburgh, United States of America

## Abstract

Chronic alcohol consumption is associated with increased incidence of ICU-related morbidity and mortality, primarily from acute respiratory distress syndrome (ARDS). However, the mechanisms involved are unknown. One explanation is that alcohol regulates epithelial sodium channels (ENaC) via oxidant signaling to promote a pro- injury environment. We used small rodent models to mimic acute and chronic alcohol consumption and tested the hypothesis that ethanol (EtOH) would affect lung fluid clearance by up-regulating ENaC activity in the lung. Fluorescence labeling of rat lung slices and in vivo mouse lung revealed an increase in ROS production in response to acute EtOH exposure. Using western blots and fluorescein-5-maleimide labeling, we conclude that EtOH exposure modifies cysteines of α-ENaC while data from single channel patch clamp analysis confirm that 0.16% EtOH increased ENaC activity in rat alveolar cells. *In vivo* lung fluid clearance demonstrated a latent increase in fluid clearance in mice receiving EtOH diet. Ethanol mice given a tracheal instillation of LPS demonstrated early lung fluid clearance compared to caloric control mice and C57Bl/6 mice. Standard biochemical techniques reveal that chronic EtOH consumption resulted in greater protein expression of the catalytic gp91^phox^ subunit and the obligate Rac1 protein. Collectively these data suggest that chronic EtOH consumption may lead to altered regulation of ENaC, contributing to a ‘pro-injury’ environment in the alcohol lung.

## Introduction

Chronic alcohol consumption is associated with an increased incidence of ICU-related morbidity and mortality, primarily due to acute respiratory distress syndrome (ARDS) [1], [2]. ARDS is a severe form of acute lung injury characterized by inflammation, edema and diffuse alveolar damage that results in impaired gas exchange, release of pro-inflammatory cytokines and the development of oxidative stress [3].

Lung edema is resolved by active salt and water transport. Epithelial sodium channels (ENaC) function to reabsorb sodium from the alveolar airway lumen and transport sodium into the cytosol where it is then extruded by the ATPase pump. Ultimately sodium reabsorption leads to the development of an electrochemical gradient between the apical and basolateral cell membranes. The electrochemical gradient facilitates the osmotically driven reabsorption of water and the subsequent resolution of lung edema.

ENaC is a multimeric protein composed of α, β and γ subunits in a fixed stoichiometry [4], [5]. ENaC activity is the rate limiting step in the resolution of lung edema. The role of ENaC in maintaining a healthy airway epithelium is highlighted by several seminal observations. First, mice lacking the α-ENaC subunit die within 40 hours of birth due to an inability to clear lung fluid [6]. Second, electrophysiology studies show that the α-ENaC subunit is vital for sodium reabsorption [7]. ENaC can be further classified into highly selective cation (HSC) channels and non-selective cation (NSC) channels according to measurements of unitary conductance and ion selectivity [8]. HSC favor Na^+^ reabsorption over K^+^ (40∶1) and have a small conductance (4–6 pS). NSC, as the name implies, are less discriminant in Na^+^ over K^+^ selectivity (1.1∶1) and have a larger unitary conductance (>12 pS) [8].

ENaC activity is regulated by reactive oxygen species [9]–[11]. However, little is known of the role of NADPH oxidase generation of oxidants in models of acute and chronic alcohol consumption (in terms of ENaC regulation and subsequent changes in lung fluid clearance). The purpose of this study was to test the hypothesis that chronic alcohol consumption would increase ENaC activity via a ROS-dependent mechanism. Recently, Bao et al has reported that pharmacological concentrations of ethanol (0.5–5% v/v) significantly increases ENaC in an ROS dependent manner using immortalized distal kidney cells [9]. In order to determine the effects of physiological doses of ethanol in the lung, we performed single channel patch clamp analysis and *in vivo* whole animal imaging to unambiguously determine the effect of ethanol on ENaC activity and subsequently lung fluid clearance and advance understanding of ROS signaling.

## Methods

### Chemicals

Ethyl alcohol (C_2_H_5_OH), 190 proof, ACS grade was purchased from Spectrum Chemical (New Brunswick, NJ) and used in all studies below. General chemicals were supplied by Sigma Aldrich (St. Louis, MO) or as indicted.

### Ethanol (EtOH) Feeding of Mice

All animal studies were performed in strict accordance with the recommendations in the Guide for the Care and Use of Laboratory Animals of the National Institutes of Health. The protocol was approved by the Emory University Institutional Animal Care and Use Committee (Permit Number: DAR-2001099 & DAR-2001310). All surgery was performed under anesthesia with xylazine and ketamine, and all efforts were made to minimize suffering. A chronic alcohol model was studied using young adult female C57Bl/6 mice purchased from Jackson Laboratory (Bar Harbor, Maine). To start, 6 week old mice were fed 5% v/v EtOH for the first week, with subsequent 5% incremental increases each week until animals reached 20% v/v EtOH. Animals remained on a 20% v/v EtOH diet for an additional 4 weeks in order to model chronic alcohol consumption. Using this general approach, the blood alcohol levels of mice following chronic ethanol consumption has been reported to be 400 mg/dL [12], [13]. An age-matched, control group of animals were fed an isocaloric maltodextrin diet. Both EtOH and control animals were given standard chow *ad libitum*; dietary intake was comparable in both groups. The benefits of using a chronic mouse model for alcohol abuse has been recently reviewed in [14].

### Acute Ethanol Treatment

Freshly isolated rat alveolar epithelial cells were treated with 0, 0.04%, 0.08%, or 0.16% v/v EtOH in a vehicle solution for 1 hour. The protocol for isolated alveolar cells has been optimized using this rodent model (see below). C57Bl6 mice ingesting 20% v/v EtOH for 3 days modeled the effects of acute alcohol consumption (i.e. binge drinking) *in vivo* and complements findings using chronic C57Bl6 mice described above.

### Primary Cell Isolation

Primary alveolar epithelial cells were isolated from male Sprague Dawley rats purchased from Charles River (Wilmington, MA) as described in [15]–[18].

### Western Blot Analysis

Whole lung homogenate was prepared in order to quantify proteins of interest from chronic alcohol mice. Briefly, mouse lung was perfused via the pulmonary artery with phosphate buffer saline (PBS). The lungs were removed *en bloc*, rinsed with PBS, and placed on ice prior to homogenization in 3 mL RIPA buffer in mM: 150 NaCl, 10 Na_3_PO_4_, 0.15 SDS, 1% Nonidet P-40, and 0.25% Na deoxycholate. Approximately 100 µg lung homogenate was electrophoresed on a 10% polyacrylamide gel in the presence or absence of DTT, as indicated. Protein was then transferred to Protran nitrocellulose membrane (Schleicher and Schuell BioScience) for immunodetection of protein of interest. Nitrocellulose was blocked in TBST buffer (10 mM Tris, pH 7.5, 70 mM NaCl, and 0.1% Tween) with 5% dry milk prior to incubation with primary antibodies of interest, as indicated. The α-ENaC-C20 antibody was purchased from Santa Cruz; antibodies for NOX2 catalytic and regulatory subunits were purchased from Millipore Upstate; and anti-Rac1 antibody was obtained from Santa Cruz. All membranes were incubated with respective antibody for 1 hr at RT, and washed extensively in TBST buffer prior to incubation with IgG alkaline phosphatase (AP) labeled secondary antibody (KPL, Inc.) at a 1∶10,000 fold dilution in TBST for 1 hr at RT. AP luminescent signal was detected using Nitro-Block chemiluminescence enhancer and CDP-Star substrate (Tropix) and Carestream Image Station Gel Logic 4000 Pro (Carestream Health) and compatible imaging software.

### Real Time PCR

Total RNA was extracted from whole lung using the RNeasy isolation kit (Qiagen) per manufacturer protocol. RNA was treated with DNase 1 and reverse transcribed using Superscript II RNaseH-reverse transcriptase (Invitrogen). The following pairs of primers were used: α-ENaC forward TGCTCCTGTCACTTCAGCAC, reverse CCCCTTGCTTAGCCTGTT C; β-ENaC forward CCCCTGATCGCATAATCCTA, reverse GCCCCAGTTGAAGATGTAGC; γ-ENaC forward ACCCTTTCATCGAAGACGTG, reverse CCTCTGTGCACTGGCTGTAA; p22^phox^ forward AACGAGCAGGCGCTGGCGTCCG, reverse GCTTGGGCTCGATGGGCGTCCACT; gp91^phox^ forward CAGGAACCTCACTTTCCATAAGAT, reverse AACGTTGAAGAGATGTGCAATTGT; p67^phox^ forward TCCAACGAGGGATGCTCTAC, reverse CACAGGCAAACAGCTTGAAC; gapdh forward CAAGGTCATCCATGACAACTTTG, reverse GGCCATCCACAGTCTTCTGG. Primer pairs were purchased from Integrated DNA Technologies (Coralville, IA). Threshold levels of mRNA expression (ΔΔCt) were normalized to mouse GAPDH levels, and values represent the mean of triplicate samples ± SEM. Data are representative of 3 independent studies.

### Single Channel Patch Clamp Analysis and Conductances

Cell-attached single channel patch clamp analysis was performed as recently described in [20]. Current-voltage relationships were plotted and chord conductances obtained by calculating the slope of points between hyperpolarizing potentials for HSC channels and between hyperpolarizing and depolarizing potentials for NSC channels. R-squared values are as indicated.

### Densimetric Evaluation of Lung Fluid Volume

We determined lung fluid volumes using an *in vivo* radiographic imaging assay recently described in [15], [16], [19]. Briefly, animals were given a tracheal instillation of saline (5 µL/g body weight) and then placed onto the platform of an *in vivo* Multispectral Imaging Station (Carestream Health) in order to determine the rate of fluid clearance in freely breathing, anesthetized mice. Animals were immobilized in the imaging station with continual delivery of isoflurane (1.5 L/min) mixed with 100% oxygen during the imaging studies. Animals were X-rayed at 5 minute intervals up to 240 min with an acquisition period of 120 sec. X-ray settings were set at 2×2 binning, 180 mm field of view, 149 µA X-ray current, 35 k Vp, and 0.4 mm aluminum filter. X-ray density was analyzed using Carestream Health MI software as described in [15], [16], [19].

### Fluorescent Reactive Oxygen Species (ROS) Detection

Three separate yet complementary approaches were used to measure ROS levels following EtOH treatment. Thin 175 micron lung slices were prepared as described in [20] prior to incubation in 10 µM dihydroethidium (DHE; Invitrogen) at 37°C for 30 min protected from light. We and others [16], [20], [21], have recently reported that fluorescent detection of the reaction product between O_2_^−^ and DHE (generating 2-hydroxyethidium) is a reliable measure of superoxide production in cells. Fluorescence detection of DHE oxidation was analyzed by confocal microscopy (excitation/emission 520/610 nm) as previously reported in [16]. DHE fluorescence intensities of lung slices were quantified using Image J, a public domain, Java-based image processing program developed at the National Institutes of Health.

200 µL DHE, or vehicle control, was tracheally instilled in C57Bl/6 lung of animals on control or acute EtOH diet, as described above. Approximately 30 min following DHE instillation, lungs were removed *en bloc*, and fluorescent detection of ROS was assayed and quantified using Carestream Health In Vivo Multispectral Imaging Station or standard plate reader with 520/625 nm excitation/emission.

Because superoxide is quickly dismutated to H_2_O_2_, we used Amplex Red Enzyme Assays (Invitrogen) as an additional measure of ROS release. Lung slices were acutely treated with EtOH. Afterwards, ROS released into the extracellular solution was incubated with Amplex Red reagent solution, prepared per manufacturer’s protocol, for 30 min at 37°C protected from light. Fluorescence intensity of Amplex Red oxidation of H_2_O_2_ was determined using a microplate reader (excitation/emission 530/590 nm).

### Immunohistochemistry and Confocal Imaging

Unfixed tissue slices were incubated in primary rabbit anti-Rac1 antibody (Santa Cruz) diluted 1∶1000, followed by incubation in secondary anti-rabbit IgG conjugated to Alexa 488 (Invitrogen) diluted 1∶50,000 in PBS containing 1% BSA and 1× sodium azide. Cell Mask Deep Red plasma membrane stain (Invitrogen) was used to mark subcellular localization of Rac1 and was used per manufacturer protocol. Lung tissue was fixed in 4% paraformaldehyde and mounted in VECTASHIELD HardSet mounting medium with 4′6′-diamidino-2-phenylindole (Vector Laboratories). Confocal imaging was conducted at the Emory-Children’s Pediatric Research Cell Imaging Core using an Olympus FV1000 confocal laser scanning microscope.

### cDNA Plasmid Transfection in Human Embryonic Kidney (HEK) Cells

Subconfluent HEK cells were cultured in DMEM/F12 50/50 media (Invitrogen) supplemented with 10% FBS, 2 mM L-glutamine, and 20 U/ml each of penicillin and streptomycin, 83.75 µM gentamycin and 1 µM dexamethasone. Cells were transfected with Lipofectamine 2000 (Invitrogen) per manufacturer’s protocol using 1 µg each α-, β-, γ- rENaC DNA plasmid constructs.

### Maleimide Labeling of Reduced Cys

Fluoroscein-5-Maleimide (F5M; Invitrogen) was used to confirm the oxidant sensitivity of lung ENaC. Briefly, endogenous α-ENaC subunit was immunoprecipitated (IPed) from male Sprague Dawley rats, chronic ethanol mice, (isolated as described in [15], [16] ) or from HEK cells over-expressing HA-tagged α-rENaC construct, alongside β-rENaC, and γ-rENaC subunits for normal channel assembly. Native protein from rat alveolar T2 cells was treated with 0, 0.04%, 0.08%, or 0.16% EtOH, whereas heterologously expressed α-ENaC was subjected to oxidation with 0, 0.1, 0.5, 1.0, and 3.0 mM H_2_O_2_. Both EtOH and H_2_O_2_ treatment were for 15 min at 37°C in a humidified 5% CO_2_ incubator. Then, 50 µL of 0.6 mM F5M (reconstituted in 20 mM Tris-HCl (pH 7.4), 0.1 mM MgCl_2_, and 1 mM MnCl_2_) was applied to 50 µL protein in standard RIPA buffer. For signal control studies, 0.6 mM F5M was pre-incubated with 6 mM glutathione. In all experiments, F5M reaction was quenched by adding equal volume 5X sample buffer containing 100 mM DTT. Samples were then boiled (95°C) for 10 min, spun down, and then electrophoresed on a 10% acrylamide gel. F5M labeled protein was transferred onto nitrocellulose and detected using an FX-Pro Multispectral Imager (Carestream Health) with 480/535 nm excitation/emission filters. Immuno-detection of α-ENaC was completed as described above. Fluorescent F5M and luminescent α-ENaC signals were contrasted, pseudo-colored, and over-laid using Carestream Health MI software.

### Evan’s Blue-labeled Albumin Measurements

C57Bl/6, maltodextrin-fed, and chronic ethanol mice were tracheally instilled with solution containing 5% bovine serum albumin (BSA) and Evans blue dye (0.1 mg/mL). Alveolar fluid clearance (AFC) was assessed 90 min after instillation by measuring the change in concentrations of alveolar Evans blue-labeled albumin. AFC was calculated as follows, and as described in [16]: AFC = [(Vi-Vf)/Vi] X 100, where Vi is the volume of instilled Evans blue-labeled albumin and Vf is the final alveolar fluid calculated as follows: Vf = (Vi X EBi)/EBf, where EB is the concentration of Evans blue-labeled albumin in the instilled solution (i) and the final alveolar fluid (f). Protein concentration was determined with the use of a Nanodrop 2000 spectrophotometer (Thermo Scientific, Wilmington, DE).

### Statistical Analysis

Data summarized as means ±SE. Single comparisons were performed using Student’s *t*-test, whereas multiple comparisons were performed using one-way ANOVA followed by Holms test for pairwise comparisons. Linear and non-linear regression analysis was performed and plotted using Sigma Plot 10 software. Post-hoc power analysis was performed using Statistical Analysis System (SAS 9.3) software, which indicated that *in vivo* sample sizes are adequate for an effect with >80% power using traditional significance criterion (alpha = 0.05).

## Results

### Acute Ethanol Treatment Increases ROS Production in the Lung

**Figure 1:**
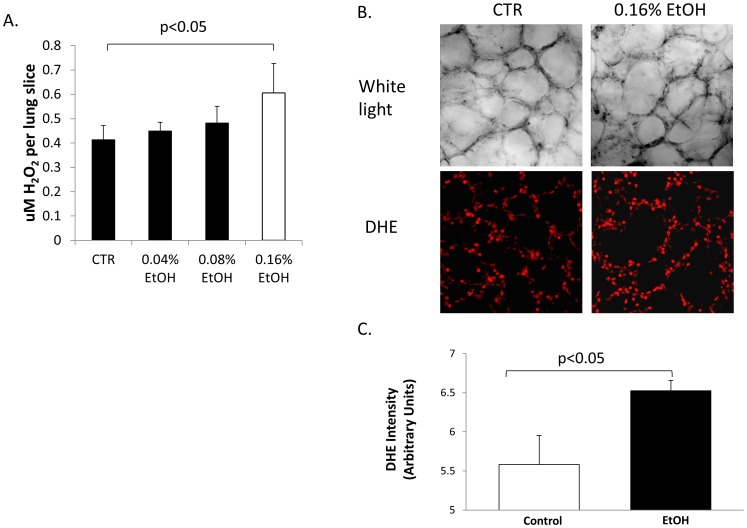
Acute EtOH treatment increases ROS production. A) Lung tissue preparation was obtained from 3 month old Sprague Dawley rats and treated acutely (for 1 hour) with various concentrations of EtOH. Amplex Red labeling indicates that 0.16% EtOH significantly increases H_2_O_2_ production. n = 9 observations from 3 animals. DHE labeling (B) and quantification (C) confirms that 0.16% EtOH significantly increases H_2_O_2_ production in lung slice preparations. n = 6 observations from 3 animals.

Ethanol-induced ROS production has been described in nervous systems as well as in alveolar macrophages [22]–[25]. In order to determine whether acute EtOH exposure can similarly induce oxidative stress in lung epithelia, we measured the levels of ROS production using Amplex Red and dihydroethidium (DHE) acutely following 0.16% EtOH application and in vivo using an acute EtOH model. Figure 1A shows that 0.16% EtOH increased the concentration of H_2_O_2_ approximately 1.3 fold compared to untreated control (n = 3, p<0.05). Confocal analysis of lung tissue slices (prepared from the middle portion of the left lobe of the lung) labeled with an alternative redox sensitive probe, DHE [21], shows spacial resolution of ROS release before (left panel) and after EtOH (right panel) treatment in the lung (Figure 1B). The top panel shows white light optical imaging of a lung slice preparation of approximately 250 microns thick. In the bottom panel, DHE labeling of ROS (610 nm red emission) was detected *in situ*. In Figure 1C, DHE fluorescence intensity was quantified with detector gain and amplifier off-set commands adjusted to the same control settings in control and EtOH fields of view using Image J software (n = 6 from 3 independent observations). Amplex Red allows for precise quantification of ROS produced, whereas DHE labeling verifies that ROS was released from alveolar epithelial cells. Taken together, Figure 1A–C confirms that acute application of 0.16% EtOH increases ROS release in the alveolar region of the lung.

**Figure 2:**
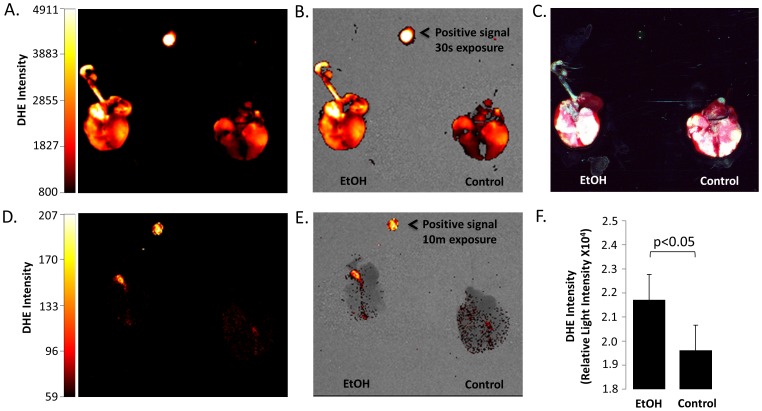
Acute EtOH consumption increases ROS production in C57/Bl6 mice. Top panels (A–C) show excised lung from control animals, or mice ingesting 20% EtOH solution for 3 days, following instillation of 200 µL DHE solution. A = 510/620 nm excitation emission (red signal); 30 sec exposure. B = panel A co-registered with x-ray image C = Additive primary color (RGB) image of lungs excised en bloc (showing trachea, heart, and lung. Panels D (510/620 nm; 10 min exposure) and E (510/620 nm co-registered with X-ray) show excised lung from control and EtOH mice following instillation of 200 µL vehicle control. Intensity bars, and rhodamine positive signal controls included for B–E. F) Quantification of homogenized lung samples (shown in A–E) using a microplate reader assay (n = 12 from 3 animals; p<0.05).

In order to verify that acute alcohol ingestion would likewise elicit an oxidative response, as opposed to direct application of EtOH, we tracheally instilled 200 µL DHE into C57Bl/6 mice maintained on a 20% v/v ethanol diet for 3 days. Figure 2A–C confirms that acute alcohol exposure increases oxidative stress *in vivo*; excised alcohol lung showed more intense DHE fluorescence signal compared to control fed animals using a 30 sec exposure time. Instilling inactivated DHE as a vehicle control showed little to no DHE fluorescence even after prolonged (10 min) exposure time in the distal portion of the lung. In order to validate and quantify DHE signal intensity, whole lung homogenate was subsequently prepared and DHE fluorescence intensities were confirmed on a microplate reader (Figure 2F).

### H_2_O_2_ and EtOH Oxidation of α-ENaC-subunit

**Figure 3:**
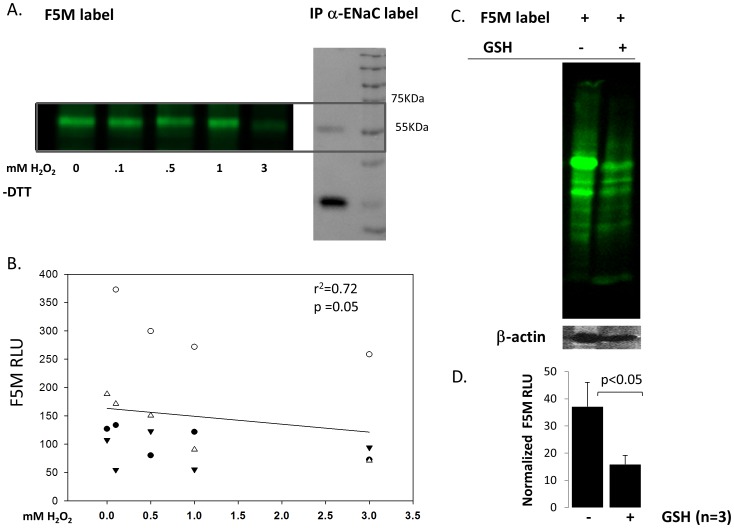
H_2_O_2_ modifies Cys thiols on α-ENaC. A) Right panel: α-ENaC protein was heterologously expressed (with β- and γ- subunits) and immunoprecipitated from HEK cells. Left panel: Fluoroscein-5-Maleimide (F5M) labeling of free Cys thiols decreases with oxidizing conditions; excitation emission 480/535 nm; non-reducing gel (−DTT). B) Simple linear regression graph of F5M relative light units (RLU) and H_2_O_2_ treatment shows relationship between Cys modification and H_2_O_2_ concentration. Dependent variable: F5M RLU. Independent variable: mM H_2_O_2_. n = 15; p = 0.05. C) Specificity of F5M label. Right lane = mouse lung homogenate labeled with 0.6 mM F5M. Left lane = mouse lung homogenate labeled with 0.6 mM F5M with/without 6 mM glutathionne. D) F5M RLU normalized to β-actin expression.

We [11], [16], and others [10], have recently reported that ROS can regulate ENaC activity and thus, influence lung and/or total body homeostasis. The precise mechanism by which ROS regulates ENaC, however, remains unclear. By labeling immuno-precipitated α-ENaC protein using fluorescein conjugated maleimides (F5M), we show direct ROS oxidation of ENaC. Generally speaking, maleimides bind to reduced –SH groups on Cys residues and α-ENaC subunits indeed have 16 highly conserved Cys residues in the extracellular loop [26]. Figure 3A shows F5M labeling of immuno-precipitated α-ENaC protein (from HEK cells heterologously expressing α-, β-, γ- ENaC subunits) in- the presence of 0, 0.1, 0.5, 1.0, and 3.0 mM H_2_0_2_. We verified the identity of F5M labeled protein using western blot analysis (Figure 3A right panel). Relative light intensities of F5M labeled α-ENaC Cys residues were plotted in Figure 2B on the y-axis with linear regression analysis showing a statistically significant correlation with changing oxidative states (x-axis); p<0.05. Figure 3C is a control study that shows specificity of F5M label for thiol groups found on cysteines. F5M labeling was performed in the presence and absence of glutathione (γ-L-glutamyl-L-cysteinyl-glycine) because the thiol group of cysteine serves as a proton donor and is responsible for the biological activity of glutathione. F5M signal, normalized to β-actin protein expression levels, show that F5M detection is significantly decreased in the presence 6 mM exogenous GSH (Figure 3C–D).

**Figure 4:**
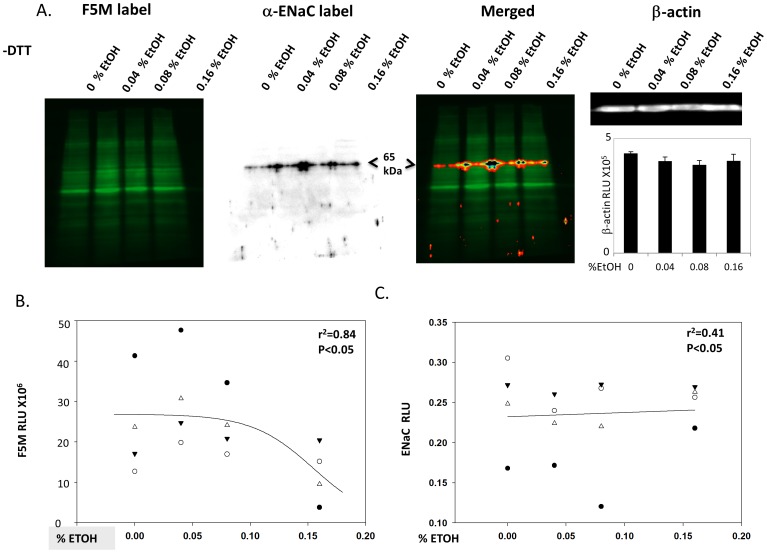
A) Acute EtOH treatment modifies Cys thiols on α-ENaC. Primary alveolar T2 epithelial cells were treated with 0, 0.4, 0.8, or 0.16% EtOH prior to F5M labeling of cells. Left panel: F5M signal intensity of EtOH treated cells; excitation emission 480/535 nm; non-reducing gel (−DTT). Middle panels: bioluminescent detection of α-ENaC subunit in F5M labeled cells using the same nitrocellulose membrane shown in left panel and anti-C 20-α-ENaC antibody (Santa Cruz) and alkaline phosphatase (AP) conjugated secondary antibody *merged* with F5M signal, as indicated. Right panel: β-actin blot and quantification shows equal amounts of protein loaded. B) Linear regression graph where F5M RLU = dependent variable and %EtOH treatment = independent variable. C) Densimetric analysis of F5M labeled primary alveolar T2 cells immunoblotted for α-ENaC.

In order to determine whether acute EtOH treatment could similarly oxidize Cys residues in lung cells, we F5M labeled alveolar T2 cells following 0, 0.4%, 0.8%, or 0.16% EtOH, as shown in Figure 4A (left most panel). The second panel identifies α-ENaC protein of interest using conventional western blot techniques and the same blot shown in the F5M labeled panel. The two signals are merged in the third panel, with the final panel indicating equal concentrations of protein loaded (based on β-actin detection). Regression plots in Figure 4B–C shows a causal relationship between EtOH treatment and α-ENaC Cys modification; as α-ENaC expression levels increase with higher EtOH concentrations (p<0.05), F5M labeling of free thiols decrease (p<0.05).

**Figure 5:**
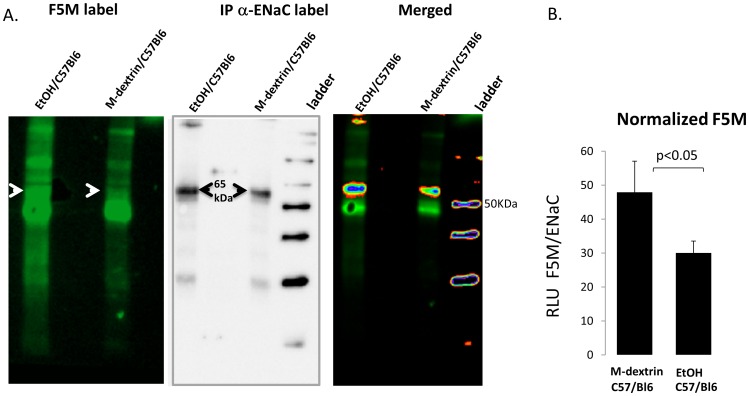
Chronic ethanol ingestion modifies Cys thiols on α-ENaC. A) Left panel F5M labeling of α-ENaC protein immuno-precipitated from chronic ethanol fed mice (left lane) or maltodextrin control animals (right lane). Middle panel shows bioluminescent detection of α-ENaC using the same membrane shown in left panel. Right panel Co-registration of F5M and ENaC signal (ENaC signal is pseudo-colored and F5M signal contrast reduced in order to enhance co-registration of data obtained from the same blot). B) Normalization of F5M RLU to ENaC expression levels; n = 3; p<0.05.

Figure 5 shows that chronic ethanol ingestion (as opposed to acute application of EtOH) can likewise modify sulfhydryl groups *in vivo*. F5M label (Figure 5A left panel) of α-ENaC subunit immuno-precipitated from C57Bl/6 mice ingesting 20% EtOH *ad libitum* for 4–6 weeks reveals that α-ENaC protein expression levels increase (middle panel) and is indeed oxidized in vivo. The arrows in Figure 5A indicate F5M labeled α-ENaC protein of interest. Co-registering the F5M fluorescent signal (merged panel), with α-ENaC antibody signal (middle panel; using the same protein blot) shows that acute ethanol ingestion alters ENaC abundance and oxidation. After normalization for α-ENaC expression levels, F5M label of ENaC is significantly decreased in EtOH lung (Figure 5B; p<0.05), indicating that acute ethanol ingestion oxidizes lung ENaC in vivo.

### Acute EtOH Treatment Increases ENaC Activity

**Figure 6:**
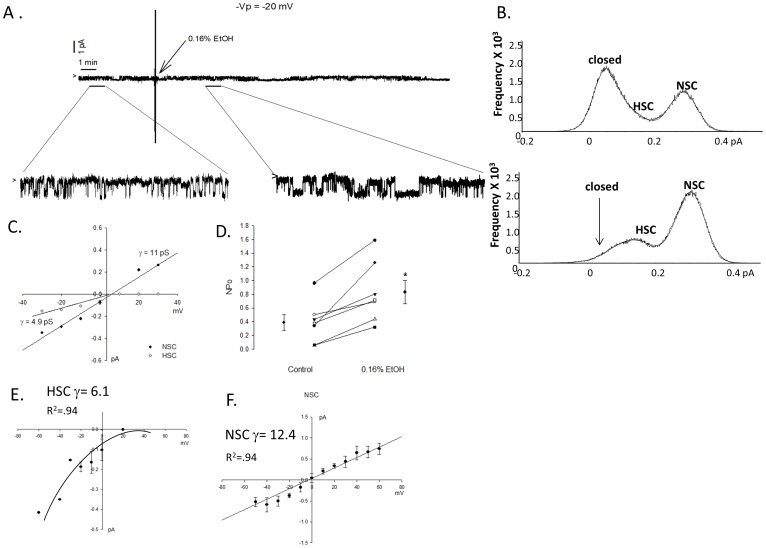
Acute 0.16% ETOH treatment increases sodium channel activity. A) Continuous single channel recording obtained from primary isolated rat T2 cell treated with 0.16% EtOH after 5 min control recording period as indicated with enlarged portions of the trace. Arrow indicates closed state of channels, with downward deflections from arrow indicating Na movement into the cell. B) Point amplitude histograms show frequency of NSC and HSC activity in representative recording before and after EtOH treatment. C) Conductances (γ) of representative HSC (4.9pS) and NSC (11pS) channels shown in representative trace. D) Number and open probability (NPo) of ENaC reported before and after 0.16% EtOH treatment in 7 cell-attached recording; p<0.05. E-F) I/V curve of all cell recordings with average HSC γ = 6.1 and NSC γ = 22.2.

If EtOH modifies post translational regulation of ENaC protein, then we expect to be able to detect putative effects on the gating properties of sodium channels expressed in the lung. Figure 6A shows a continuous cell-attached patch clamp recording obtained from a primary alveolar T2 cell. Enlarged portions of the trace, and point amplitude histograms show that 0.16% EtOH treatment increases ENaC activity (Figure 6B; with representative conductances shown in 6C). While Figure 6A–B is a representative recording, EtOH indeed increased ENaC NPo in all recordings obtained (summarized in Figure 6D). Calculated NPo values represent the net effect of EtOH on both HSC and NSC activity. Specifically, all cells expressed NSC activity, with 5 out of the 7 observations expressing both HSC and NSC (average conductance shown in Figure 6E–F).

### Chronic EtOH Ingestion Increases α-ENaC and Nadph Oxidase (NOX) Protein Expression

**Figure 7:**
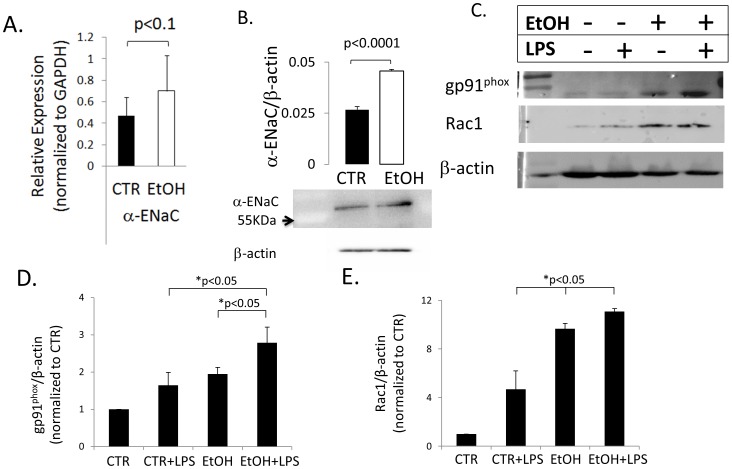
Chronic ethanol ingestion alters protein expression in the lung. A) Normalized α-ENaC mRNA levels are elevated in C57Bl/6 mouse lung chronically ingesting 20% v/v EtOH. Data represents 9 independent observations from 3 animals in EtOH and isocaloric control groups. B) Expression of α-ENaC protein is significantly increased in mice chronically ingesting 20% w/v EtOH. Ave of data reported from n = 3 mice from EtOH and isocaloric control groups. Data normalized to β-actin expression levels, and p<0.0001. C) Representative western blot of C57Bl/6 mouse lung following chronic alcohol, or isocaloric control diets w/without 1 mg/mL LPS inoculation. D,E) Densimetric analysis show NOX2 catalytic domain gp91^phox^ (n = 3) and Rac1 (n = 6) significantly increases following (chronic) EtOH and (acute) LPS treatment. p<0.05 where indicated by asterisks.

Oxidative stress and altered protein expression has been reported in murine models of chronic ethanol consumption [27]–[29]. Figure 7A–B shows changes in α-ENaC transcript levels in a mouse model for chronic alcohol consumption, which lead to significant increases in the expression level of α-ENaC protein in similarly treated mice chronically consuming EtOH up to 12 weeks. Figure 7B normalizes for β-actin expression levels, indicating that averaged densimetric values of α-ENaC protein levels increased nearly 2-fold (n = 4) after normalization. In the representative western blot shown in 7B, EtOH increased the densimetric determination of α-ENaC protein levels by 18% (again, after normalization using β-actin). The increase in ENaC expression in chronic EtOH animals are in-line with the increase in sodium channel subunit detection levels first presented in Figures 4 and 5. In Figure 7C–E, we evaluated the effects of chronic EtOH ingestion on the catalytic domain of NOX2 (gp91^phox^) and small g protein Rac1. Given that our laboratory has recently reported that Rac1-control of NOX enzymatic activity, and hence ROS release (causing up-regulation of ENaC activity [30]), these NOX proteins are of particular interest. In a mouse model of chronic alcohol ingestion, gp91^phox^ and Rac1 expression significantly increased in T2 cells following 1 mg/mL LPS (p<0.05 vs. CTR, CTR+ LPS, EtOH animals, Figure 7D–E).

**Figure 8:**
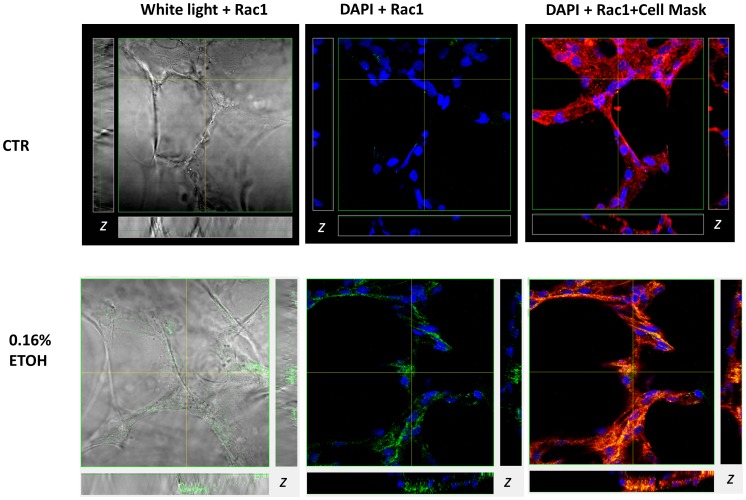
Multichannel confocal imaging shows EtOH mediated changes in Rac1 expression and subcellular localization in the lung. Anti-Rac1 antibody detected using Alexa 488 conjugated secondary antibody (488/519 nm; green fluorescence); nuclei were stained DAPI (350/470 nm; blue fluorescence); and plasma membrane labeled with Cell Mask Deep Red (649/666 nm). Right panel: white light image of lung slice preparation and Rac1 labeling; middle panel: Rac1 localization relative to DAPI stained nuclei; left panel: Rac1 co-localization with Deep Red labeled plasma membrane. Pixels containing both red and green color contributions produce various shades of orange and yellow indicate Rac1 co-localization with the plasma membrane. Subsets represent z-axis obtained from horizontal and vertical regions as indicated.

Upon activation, Rac1 is known to translocate and interact directly with the N-terminal region of Nadph oxidase [31]. We therefore examined whether Rac1 translocates to the apical membrane of alveolar cells, where gp91^phox^ expression has indeed been reported [20], following acute EtOH treatment. In Figure 8A, the left panel shows white light illumination of a lung slice preparation with alveolar cells intact. The cell free areas are alveolar (airspace) that had been inflated by instilling agarose in order to stabilize the tissue without paraffin or optimal freezing compound, as described in [20]. Rac1 labeling was performed in live lung tissue preparations in the presence and absence of 0.16% EtOH (middle panel). Likewise, Cell Mask Plasma membrane labeling was performed before fixation (right panel); and DAPI labeling of the nuclei (blue signals) was performed after fixation and alongside mounting slides. Z-sections of confocal images (shown as side panels on the vertical and horizontal planes) shows both increased Rac1 expression (middle panel) and translocation to plasma membrane (right panel) following 0.16% EtOH treatment.

### LPS Accelerates the Rate of Alveolar Fluid Clearance via Rac1 Signaling in EtOH Mice

**Figure 9:**
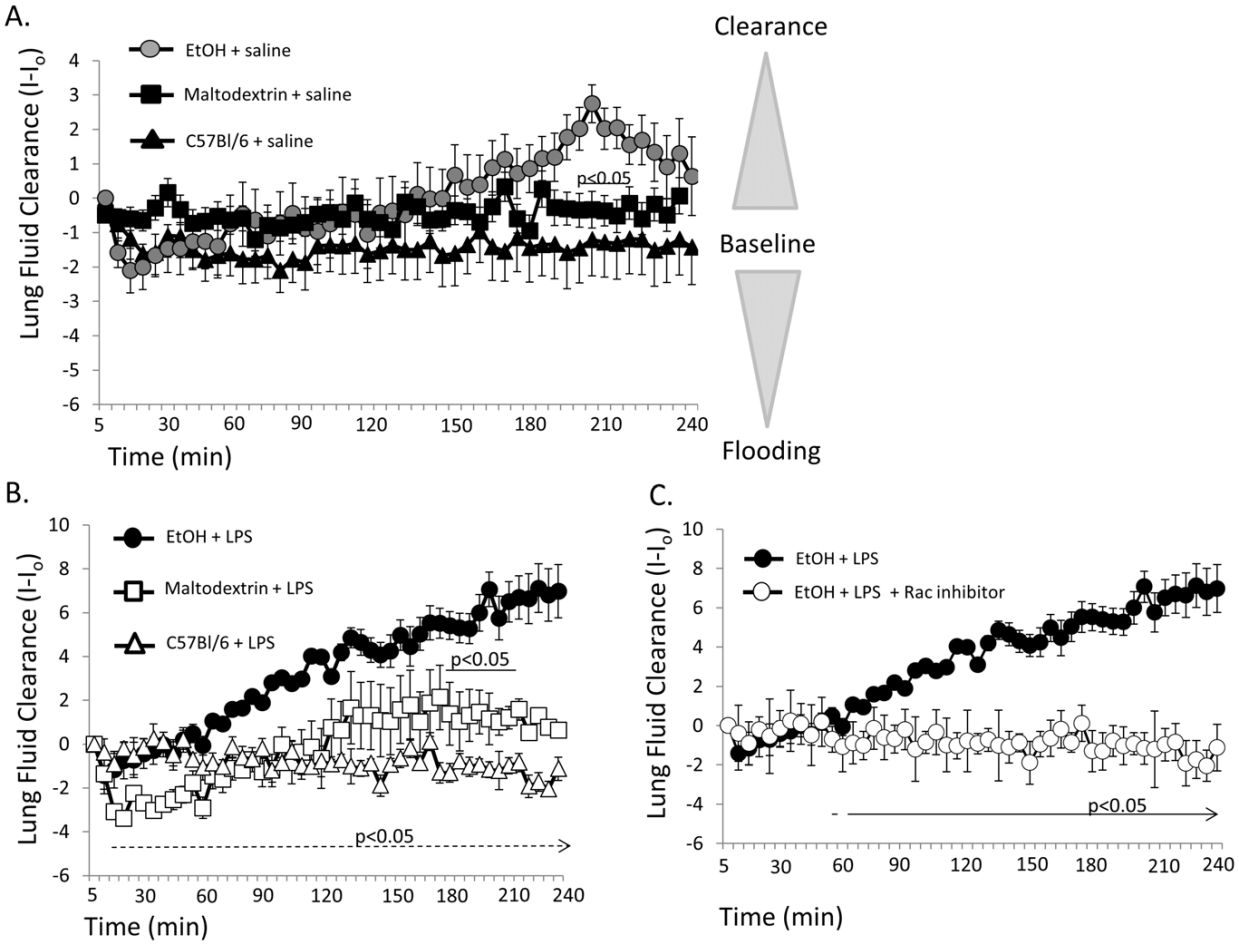
LPS inoculation and chronic EtOH ingestion enhances the rate of alveolar fluid clearance. A) In vivo assessment of lung fluid volumes after saline challenge in EtOH (n = 13), isocaloric control (maltodextrin, n = 13) and C57Bl/6 mice (n = 10) indicate similar patterns of lung fluid clearance; albeit an observed delay in clearance in EtOH mice compared to maltodextrin between 3.5–4 hrs following saline challenge. B) In vivo assessment of 1 mg/mL LPS inoculated animals chronically fed an EtOH diet (n = 4) cleared fluid at faster rates compared to isocaloric control groups of animals fed maltodextrin (n = 5) between 190–220 min following saline challenge (as indicated by solid line, p<0.05). LPS inoculation of EtOH animals showed significantly elevated rates of alveolar fluid clearance compared to LPS inoculated C57Bl/6 mice beginning at 20 minutes following saline challenge (denoted by dashed line, p<0.05). C) Co-instillation of 1 µM NSC23766 and LPS in chronic ethanol mice indicates that small G protein Rac1 plays an important role in alcohol lung and alveolar fluid balance. Animals instilled with LPS and NSC23766 (n = 5) failed to clear saline challenge to the extent observed in LPS (n = 4) inoculated EtOH animals; p<0.05 as indicated.

Our laboratory has recently developed a new fluorometric (X-ray) approach to determining lung fluid volume [15], [16], which has been validated against conventional wet:dry lung weight measurements and the Evan’s blue assay useful in determining alveolar fluid clearance. In Figure 9A, we evaluated changes in real time X-ray images over a 4 hour period (time on x-axis) of freely breathing anesthetized mice following a saline challenge of 5 µL/g mouse. Radio-opacities of X-rayed lungs were quantified, and then normalized to initial X-ray image following saline challenge (expressed as I-I_o_ on y axis; where decreases in I-I_o_ values indicate fluid movement into the lung and more positive values indicate alveolar clearance). *In vivo* determination of lung fluid volumes indicate that animals chronically ingesting EtOH have slight increases in the ability to clear saline challenges, particularly at the 190–220 min time points of evaluation where p<0.05 compared to caloric control groups of animals. There was no difference in fluid clearance between caloric controls and the normal fed C57Bl/6 mouse. In Figure 9B, we compared fluid clearance in mice inoculated with LPS. LPS inoculation (1 mg/mL) significantly increased fluid clearance in EtOH mice compared to caloric (maltodextrin) and normal fed C57Bl/6 mice inoculated with LPS. In Figure 9C, we show that LPS failed to induce robust changes in alveolar fluid clearance in the presence of 1 µM Rac inhibitor, NSC23766; thus implicating small G protein Rac1 signaling in LPS and EtOH mediated pathways.

### Evan’s Blue Dye as a Marker for Lung Fluid Clearance Validates Fluorometric Evaluation of Lung Fluid Volume Obtained in vivo

**Figure 10:**
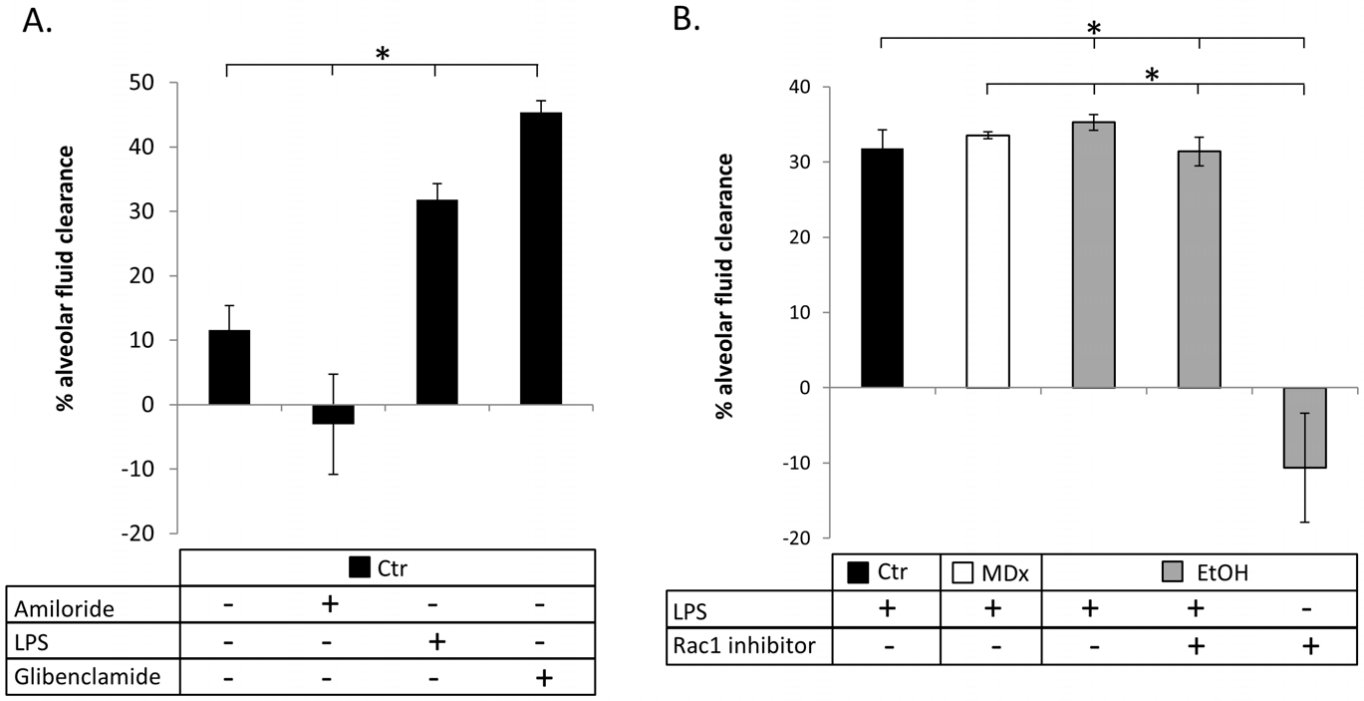
Changes in albumin protein concentration confirm fluorometric data obtained from live animals. A) C57Bl6 mice challenged by IT instillation of saline, 1 mM amiloride, 1 mg/mL LPS, or 1 mM glibenclamide as indicated. n = 2 for each observation with * = p<0.05. B) C57Bl6 mice (black bars), maltodextrin-control fed mice (white bars); or chronic ethanol mice (grey bars) were treated with 1 mg/mL LPS in the presence or absence of 1 µM NSC23766. n = 3 independent observations with * = p<0.05. In 10A–B, the percent changes in albumin protein concentration were obtained 90 min following instillation; percentages greater than average control values indicate enhanced rates of fluid clearance, whereas negative percent changes are indicative of alveolar flooding.

Figure 10A detects changes in albumin protein concentration as a measure of alveolar clearance. Using this approach, we verify that alveolar fluid clearance is indeed inhibited by amiloride, and enhanced by LPS and chloride channel inhibitor, glibenclamide, as has been established in [16], [32], [33]. The novel observation made using this conventional approach (shown in 10A–B) is that LPS increases the rate of alveolar fluid clearance in each group of animal study (normal fed; maltodextrin fed; or chronic EtOH fed; p<0.05). Co-inoculation of LPS and small G protein Rac1 inhibitor compound NSC23766, however, significantly decreased lung fluid clearance (to approximately control levels using Evan’s blue dye). NSC23766 (alone) instillation into chronic alcohol lung lead to severe alveolar flooding compared to all groups of animals evaluated in Figure 10B using Evan’s blue dye. In summary, Figure 10A–B validates the real-time lung fluid clearance data reported in Figure 9 (that LPS stimulates lung fluid clearance in chronic ethanol mice via Rac1 signaling) using Evan’s blue-labeled albumin measurements.

## Discussion

Major findings of this study include that ethanol increases NADPH oxidases regulation of epithelial sodium channels. This observation, and others herein, have potential clinical implications and warrant additional discussion.

### Acute and Chronic Ethanol Exposures

We applied various (low) concentrations of ethanol in our in vitro experiments (Figure 1). It has been previously reported that alcohol is absorbed and transported in an unbound and unaltered state throughout the body [34]. Highly vascularized organs, such as the lungs, achieve the highest concentration of alcohol, and because ethanol is not immediately metabolized, ethanol concentrations from exhaled breath can be measured and changes quantified [35]. This is, in fact, the premise behind breathalyzers and studies have shown a strong correlation between alcohol levels from serum and exhaled breath [36], [37]. In this study we also applied ethanol in concentrations that exceed the legal limit of intoxication to demonstrate the effects of acute alcohol exposure.

Generally speaking, we examined the consequence of acute ethanol exposure and ingestion, as well as chronic ethanol ingestion in rat and mouse lung. Regardless of the length of EtOH exposure and animal model, there is a clear effect on oxidative stress and up-regulation of Na channel activity observed. Specifically, F5M labeling of reduced Cys shows that ENaC is oxidized following H_2_0_2_ exposure (Figure 3); acute 0.16% EtOH exposure (Figure 4); and following chronic ethanol ingestion (Figure 5). Moreover, the aforementioned figures, together with Figure 7, shows that α-ENaC subunit protein consistently increases following acute and chronic EtOH exposure. Our observation of enhanced ENaC activity, following acute exposure to 0.16% EtOH treatment is in-line with previous published reports of chronic ethanol ingestion and net Na transport in alveolar type 2 cells. We observed a clear increase in ENaC activity following acute 0.16% EtOH treatment with the preponderance of channels being NSC channels. The increase in NSC activity in our studies are in-line with the previous observation reporting that the predominant sodium-permeant channel seen in apical membrane patches of type 2 cells obtained from rats maintained on a chronic Lieber-DeCarlie liquid diet (with 36% total calories from EtOH) were the high-conducting NSCs (γ = 20.6±1.1pS) [38]. Moreover, confirmatory studies performed in an immortalized epithelial cell line, reported by Bao et al, indicates that acute application of EtOH (5% or 0.5%), and acetaldehyde (1%) likewise increases ENaC NPo in an ROS dependent manner. The aforementioned study also reports an increase in biotinylated α-ENaC expression following ethanol treatment in kidney epithelia. Coupled with our F5M labeling of α-ENaC presented in Figures 3, 4, and 5, there is intriguing possibility that EtOH (via ROS production and signaling) increases active α-ENaC at the cell surface via Cys modification. This is indeed an area of continued research interest.

Whether acute exposure to EtOH predisposes the lung to ARDS as chronic alcohol does, remains unclear; however, support from the literature indicates that both acute and chronic ethanol exposure may significantly alter the lungs’ ability to heal. For example, Li and colleagues demonstrated in a rat burn model that acute exposure to blood alcohol of 0.1% increased both neutrophil migration into the alveolar space and edema [39]. Thus, suggesting that acute ethanol affects the lung.

### Sodium Channel Activity and Rate of Alveolar Fluid Clearance

The effect of acute and chronic EtOH exposure on NSC activity may explain the latent changes seen in lung fluid clearance following a tracheal instillation of saline (as shown in Figure 9A). Given that NSCs are less efficient in generating osmotic gradients (because of its nearly equal permeability ratio for Na and K ions) a lag time between NSC activation and net alveolar fluid clearance would be expected. In fact, this observation has been reported for activation of NSC channels by denopamine in alveolar type 1 and type 2 cells. Intraperitoneal injection of denopamine resulted in an approximate 2 hour delay in lung fluid clearance, compared to IP injection of terbutaline (which activated HSC channels in alveolar cells and immediately cleared the instilled saline within 20 minutes) [40]. Likewise, LPS activation of a 20 pS NSC in type 1 cells resulted in significant changes in lung fluid volumes after approximately 3–4 hours following a saline challenge [41]. Thus, the NSC ENaC is temporally less efficient at removing excess lung fluid. The combination of our electrophysiology measurements and *in vivo* lung fluid clearance data allow for the stratification of HSC and NSC effects in the lung which has not been previously possible.

### Ethanol Regulates α-ENaC Subunit Expression and Oxidation

Classically, α-, β-, and γ-ENaC subunits comprise epithelial sodium channels. In this study, we show that chronic ethanol increases α-ENaC protein expression and thiol modification in-part using an anti-α-ENaC antibody (commercially available from Santa Cruz) that recognizes the C-terminal domain of α-ENaC. Many groups have reported that this antibody recognizes the cleaved 65 kDa fragment of ENaC, and moreover, that the 65 kDa band is the active form of α-ENaC [42]–[45]. The importance of this is highlighted by the observation that expression of α-ENaC alone produces current, and that the α-ENaC channel phenotype is predominantly an NSC [46].

### Nadph Oxidase and Lung Injury in Alcohol Mice

In our studies, we show that a secondary insult, mimicked by a tracheal instillation of LPS, significantly increased the expression of the catalytic gp91^phox^ subunit, as well as the small g-protein, Rac1 in the ethanol animals (Figure 5C–E). Rac-1 interacts with the Nox2 regulator subunits and translocates them to the catalytic domain to facilitate the conversion of molecular oxygen (O_2_) to the free radical superoxide (O_2_^−^). LPS instillation in ethanol mice significantly increased lung fluid clearance, an effect that was observed early (20–25 minutes) and persisted throughout the 4 hour experimental protocol (Figure 7). We have previously reported that superoxide regulates ENaC activity [16], [30] and given the increase in protein expression for Rac-1 and gp91^phox^ in the lung tissue of chronically fed ethanol mice, it is reasonable to suggest that chronic ethanol consumption leads to increased Nox2 protein expression. An increase in Nox2 protein suggests that the lung is “primed” to respond. However, if chronic ethanol consumption “primes” Nox2 production which can positively regulate ENaC to remove lung fluid, why are alcoholics admitted to the ICU more likely to develop severe ARDS and die?

The answer is not known. However, based on findings from other studies it seems likely that chronic alcoholic consumption could lead to dehydration of the epithelial lining fluid (ELF), the antioxidant rich fluid which coats the alveolar epithelium and is essential for effective gas and ion exchange. Others have described a cystic fibrosis-like phenotype in the airway when the ELF is dehydrated, and accompanying the dehydrated airway there is thick, dehydrated mucous that can lead to the development of spontaneous infection of the lower airways, and chronic alcohol abuse has been shown to decrease airway responsiveness [47]–[50]. Additionally, chronic alcohol consumption may alter the immune response which may result in increased or aberrant phagocytic activity and greater levels of ROS [28], [29], [49]. Last, it is important to consider the source of ARDS in alcoholics, as aspiration pneumonia will result not only in airway inoculation with gram negative organisms (which express LPS on their cell walls) but also can lead to chemical injury and pneumonitis. Together with altered regulation of Nox, ENaC acting against this background may lead to poorer outcomes in critically-ill alcoholics with ARDS.
