# Correction: Ethanol Alters Alveolar Fluid Balance via Nadph Oxidase (NOX) Signaling to Epithelial Sodium Channels (ENaC) in the Lung

**DOI:** 10.1371/annotation/9ebb4691-e5ae-4cf8-a368-c15a684a2873

**Published:** 2013-02-12

**Authors:** Charles A. Downs, David Q. Trac, Lisa H. Kreiner, Amity F. Eaton, Nicholle M. Johnson, Lou Ann Brown, My N. Helms

Author My N. Helms should not have been given Equal Contribution. The two Equally Contributing first authors are Charles A. Downs and David Q. Trac. 

